# JNK1 Signaling Downstream of the EGFR Pathway Contributes to Aldara^®^-Induced Skin Inflammation

**DOI:** 10.3389/fimmu.2020.604785

**Published:** 2021-02-05

**Authors:** Aurore Le, Abdulkader Azouz, Séverine Thomas, Nicolas Istaces, Muriel Nguyen, Stanislas Goriely

**Affiliations:** Institute for Medical Immunology and ULB Center for Research in Immunology (U-CRI), Université Libre de Bruxelles, Gosselies, Belgium

**Keywords:** JNK1, EGFR, imiquimod, skin, inflammation, psoriasis, Aldara

## Abstract

c-Jun N-terminal protein kinase 1 (JNK1) is involved in multiple biological processes but its implication in inflammatory skin diseases is still poorly defined. Herein, we studied the role of JNK1 in the context of Aldara^®^-induced skin inflammation. We observed that constitutive ablation of JNK1 reduced Aldara^®^-induced acanthosis and expression of inflammatory markers. Conditional deletion of JNK1 in myeloid cells led to reduced skin inflammation, a finding that was associated with impaired Aldara^®^-induced inflammasome activation *in vitro*. Next, we evaluated the specific role of JNK1 in epidermal cells. We observed reduced Aldara^®^-induced acanthosis despite similar levels of inflammatory markers. Transcriptomic and epigenomic analysis of keratinocytes revealed the potential involvement of JNK1 in the EGFR signaling pathway. Finally, we show that inhibition of the EGFR pathway reduced Aldara^®^-induced acanthosis. Taken together, these data indicate that JNK1 plays a dual role in the context of psoriasis by regulating the production of inflammatory cytokines by myeloid cells and the sensitivity of keratinocytes to EGFR ligands. These results suggest that JNK1 could represent a valuable therapeutic target in the context of psoriasis.

## Introduction

Psoriasis is a chronic disease of unsolved pathogenesis that affects skin and joints in 1–3% of the general population. It arises in genetically susceptible hosts in response to ill-defined environmental triggers and is characterized by inflamed and scaly skin lesions and can be complicated by arthritis (1). The skin lesions show hyperproliferation and altered differentiation of epidermal keratinocytes, marked infiltrates of T cells and neutrophils, and a distinct increase of skin capillaries (2). While psoriasis is considered as an autoimmune disease, the current pathogenic model emphasizes the role of various innate immune populations (e.g., dermal dendritic cells, neutrophils, or γδ T cells) and the IL-23/IL-17 axis. It is clear however that keratinocytes also contribute to the initiation of inflammation. IL-17 stimulates keratinocytes and dermal fibroblasts leading to the expression of multiple antimicrobial peptides and chemokines that recruit and activate inflammatory cells (3). Both TNF and IL-23/IL-17 axes now represent major therapeutic targets that have dramatically changed the management of psoriasis and psoriatic arthritis (2).

c-Jun N-terminal protein kinase 1 (JNK1), encoded by *Mapk8*, is a member of the mitogen-activated protein kinase (MAPK) family and is involved in many processes such as embryonic development, neuronal functions, cancer, metabolic inflammation, antimicrobial defense or immune-related signaling cascades (4). Its role in the control of skin homeostasis has not been fully elucidated although it was shown to contribute to epidermal development (5). Recently, a heterozygous splice site mutation in *MAPK8* in three affected members from a multiplex family with autosomal dominant chronic mucocutaneous candidiasis disease was reported. The mutation results in aberrant splicing of *MAPK8* mRNA and reduced expression of JNK1 (6). As the same cytokine network is involved in psoriasis and skin defense against fungal pathogens such as *C. albicans* (7), we hypothesized that JNK1 could participate in the pathogenesis of psoriasis. We therefore evaluated the role of this kinase in the context of Aldara^®^-induced skin inflammation, a widely used *in vivo* model of psoriasis to study the interactions between immune cells and keratinocytes (8). We demonstrate that in this context, JNK1 plays a dual role: On one hand, it acts in myeloid cells, leading to inflammasome-related inflammation and on the other hand, it contributes to keratinocyte proliferation downstream of the EGFR pathway.

## Material and Methods

### Mice

All experiments were performed on age-matched (from 8 to 12 weeks of age) female mice. Wild type C57Bl/6 mice were purchased from Envigo. *Mapk8^fl/fl^* mice (C57BL/6 background) were previously described (9) and kindly provided by Thomas Wunderlich and Jens Brüning, Institute for Genetics, University of Cologne. B6.C-Tg(Pgk1-cre)1Lni/CrsJ (stock 020811), B6.129P2-Lyz2tm1(cre)Ifo/J (stock 004781), B6N.Cg-Tg(KRT14-cre)1Amc/J (stock 018964) and B6.Cg-Tg(Itgax-cre)1-1Reiz/J (stock 008068) mice were obtained from the Jackson Lab. Littermates were used as controls in all experiments. All mice were bred and maintained in a conventional animal facility.

### Aldara^®^-Induced Dermatitis and Recombinant Cytokine Injection

Eight- to 12-week-old female mice were shaved on the abdomen with an electrical shaver and depilated with Veet hair remover (10). Mice were topically treated with a 62.5 mg/day of cream containing 5% imiquimod (Aldara cream^®^, 3M pharmaceuticals) over eight consecutive days (11). Mice were sacrificed 4 h after the last application, and skin samples were collected for histology, gene expression or tissue processing. Carrier-free rmIL17A (R&D Systems, #7956ML025/CF) 500 ng/ear/day were injected daily for 8 days, then mice were sacrificed and ears were sampled for histology. Carrier-free rmIL23 (BioLegend, #589006) 1μg/ear/day or carrier-free rmAREG (PeproTech, #31536) 1μg/ear/day were injected daily for 4 days, then mice were sacrificed and ears were sampled for histology. When indicated, SP600125 30 mg/kg/d (VWR), AG1478 15mg/kg/d (VWR) or DMSO was injected intraperitoneally to mice, once per day for 9 days.

### Cell Preparation for Keratinocyte Cell Sorting

Skin samples were incubated with dispase II (Sigma-Aldrich, 2.4 mg/ml), collagenase IV (Worthington, 0.4 mg/ml) and DNase I (Sigma-Aldrich, 100 μg/ml) for 2h at 37°C. Dead cells were stained with LIVE/DEAD™ Fixable NearIR Stain Kit, for 633/635 nm excitation (Life Technologies). Then, they were incubated with rat anti-mouse CD16/CD32 (BD, 2.4G2, dilution 1:100, 553141), and a surface staining antibody mix CD45-PE (BD, 30F11, dilution 1:100, 553081), CD31-PE (BD, MEC 13.3, dilution 1:100, 561073), CD140a-PE (BD, APA5, dilution 1:100, 562776), EpCam-BV421 (BD, G8.8, dilution 1:100, 563214), and CD49f-PerCpCy5.5 (BD, GoH3, dilution1:100, 562495). Cells were sorted on a BD FACSAria™ III.

### RNA Purification and RNA Sequencing

Keratinocytes RNA from WT or *Mapk8^∂EP^* mice was extracted with the RNeasy Minikit (Qiagen) and sent for RNA sequencing. Libraries were prepared using Ovation SoLo RNA-Seq System

(NuGEN Technologies) and underwent paired-end sequencing (25 Å~ 106 paired-end reads/sample, NovaSeq 6000 platform) performed by BRIGHTcore ULB-VUB, Belgium (http://www.brightcore.be). Adapters were removed with Trimmomatic-0.36 (with the following parameters: Truseq3-PE.fa:2:30:10 LEADING:3 TRAILING:3 SLIDINGWINDOW:4:15 MINLEN:36 HEADCROP:4) Reads were then mapped to the reference genome mm10 by using STAR_2.5.3 software with default parameters. We then sorted the reads from the alignment according to chromosome positions and indexed the resulting BAM files. Read counts in the alignment BAM files that overlap with the gene features were obtained using HTSeq-0.9.1 with “nonunique all” option (if the read pair aligns to more than one location in the reference genome, it is counted in all features to which it was assigned and scored multiple times). Genes with no raw read count greater than or equal to 20 in at least one sample were filtered out with an R script, raw read counts were normalized, and a differential expression analysis was performed with DESeq2 by applying an adjusted P < 0.05 and an absolute log2 ratio larger than 1. Gene Set Enrichment Analysis (GSEA) was performed on the keratinocytes dataset to examine the enriched gene ontology terms. Resulted pathways were introduced to Cytoscape to generate an enrichment map for functional enrichment visualization.

### ATAC Sequencing

After sorting, 20,000–50,000 keratinocytes were centrifuged, washed once with icecold PBS and resuspended in 50 μl of lysis buffer (10 mM Tris-HCl, 10 mM NaCl, 3 mM MgCl2, and 0.1% IGEPAL). Cell suspension was directly centrifuged (500 g) for 10 min at 4°C. Supernatant was discarded and nuclei were resuspended in 50 μl of reaction buffer (2.5 μl of Tn5 transposase, 22.5 μl of TD buffer, and 25 μl of H_2_O, Nextera DNA sample preparation kit, Illumina). The reaction was performed for 30 min at 37°C. DNA was purified using the MinElute PCR Purification Kit (QIAGEN), amplified, and indexed by PCR using NEBNext High-Fidelity 2 Å~ PCR Master Mix (New England Biolabs) with 10–12 cycles. Amplified libraries were purified using MinElute PCR Purification Kit (Qiagen) and quality controlled using a Bioanalyzer High-Sensitivity DNA Analysis kit (Agilent). Paired-end sequencing was performed on NovaSeq platforms (Illumina). Paired-end reads were mapped to mouse genome mm10 with Bowtie2 using default parameters. Reads that mapped several regions, or with insufficient mapping quality, were removed with samtools view. Peaks were called with MACS249 using the following parameters: -f BAMPE -g mm -q 0.05 –nomodel –call-summits -B –SPMR. We created an atlas containing all obtained peaks for all the populations using bedtools with a minimum overlapping of 1 bp. The obtained atlas was subjected to differential analysis using DESeq2 (p-adjusted cutoff of 0.05) provided by SeqMonk 1.43.0 (Mapped Sequence Analysis Tool, Babraham Bioinformatics, http://www.bioinformatics.babraham.ac.uk/projects/seqmonk/). Resulting peaks were separated into two categories: peaks located in promoters (located within 2 kb around the transcription start site) and peaks located in enhancers (not located in the defined promoter regions). For downstream visualization, a scaling factor was calculated using deepTools package to normalize peak intensity to fraction of reads in peaks (FrIP) and generate bigWig files. Gene Ontology analysis was performed by introducing BED files from differential ATAC-Seq peaks to GREAT with default parameters. For motif analysis, CiiiDER algorithm was used to perform motif enrichment in the differentially accessible regions.

### Gene Expression

Total skin RNA was extracted with the NucleoSpin RNA plus (Filter service, catalog MN 740984.250) and was reverse-transcribed with the RevertAid RT Reverse Transcription Kit (Thermo Fisher Scientific, catalog K1691). cDNA was amplified using TaqMan probes or SYBR green.

Relative mRNA levels were determined by comparing the cycle thresholds for the gene of interest and a calibrator gene *Actb*, then values for Aldara^®^-treated group were compared with mock-treated group and finally all values were compared to the median value of *Mapk8^fl/fl^* or *Mapk8^+/+^* Aldara^®^-treated group arbitrarily set at the value of 100. Primers and probes sequences are available in **Supplemental Material Table 1**.

### Histology

Mice skins were fixed in 4% paraformaldehyde. Samples were directly paraffin embedded. Sections (4 μm) were stained with May Grünwald Giemsa, hematoxylin eosin or Ki67. Epidermal thickness was measured at 200-fold magnification; a mean of three measures on three different slide fields was calculated for each sample by two-blinded observers. Ki67 epidermal positive cells were quantified manually at 400-fold magnification; a mean of three counts on three different slide fields was calculated for each sample by two-blinded observers.

### Cell Culture

Primary keratinocytes were isolated from newborn mice (24–72 hours after birth) (Li 2013). Cells were cultured in complete Keratinocyte Growth Medium II (Promocell GmbH) at 7% CO2 and 36°C.

Bone marrow-derived macrophages (BMM) were generated as previously described (12). BMM were stimulated with Aldara cream^®^ (250 mg of Aldara^®^ cream diluted in 1.5ml of DMSO, 3M pharmaceuticals), R837 (Invivogen #tlrlimqs) or R848 (Invivogen, #tlrlr848) or primed for 3h by UltraPure lipopolysaccharide (LPS from E. Coli 0111:B4, Invivogen #tlrl3pelps). TNF, CXCL1, IL-12p40, and IL-1β were measured in supernatant by ELISA (R&D Systems).

Bone-marrow derived dendritic cells (BMDC) were generated as previously described (12).

### EDU Staining

Cells were cultured in complete Keratinocyte Growth Medium II (Promocell GmbH) at 7% CO2 and 36°C. 647 EdU Click Proliferation Kit (BD, #565456) was used according to datasheet and dead cells were stained with LIVE/DEAD™ Fixable NearIR Stain Kit, for 633/635 nm excitation (Life Technologies). Data were acquired on a BD LSRII Fortessa and analyzed with Flowjo software.

### Western Blotting

Mouse skin and spleen samples were lysed in RIPA reagent and treated like previously described (10). Mouse anti-GAPDH (clone 6C5; Meridian Life Science) and mouse anti-JNK1 (F-3; SC1648; Santa Cruz) were used as primary antibodies.

### Statistical Analysis

Results are expressed as median ± interquartile range. Statistical significance was assessed using Mann-Whitney test and results were considered significant at p < 0.05. (GraphPad Prism 6.0).

## Results

### JNK1 Contributes to Aldara^®^-Induced Skin Inflammation

**Figure 1:**
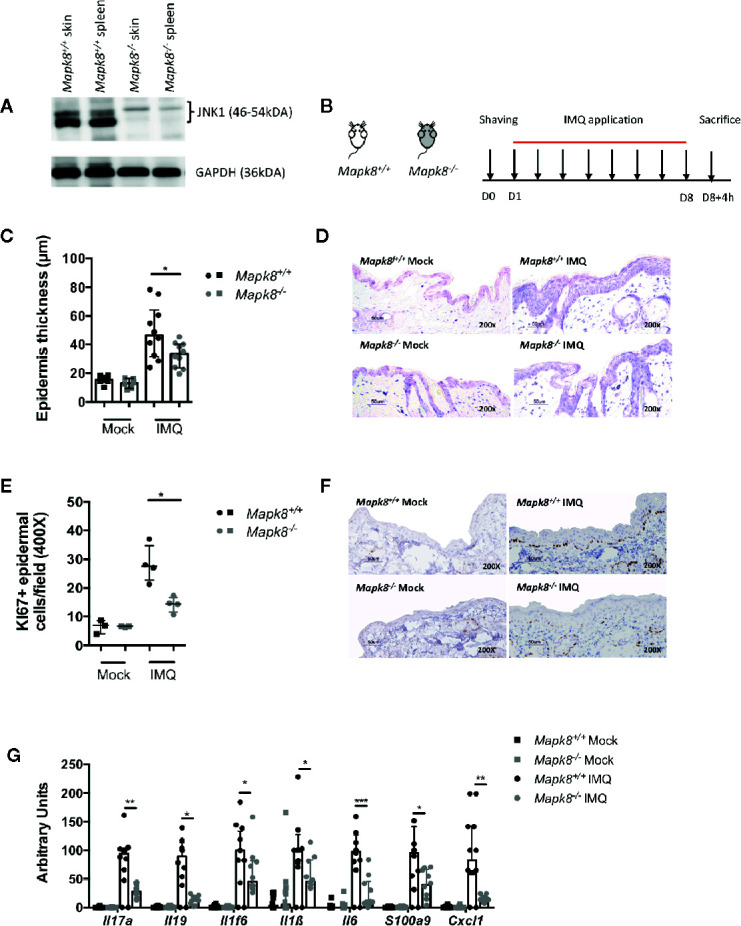
JNK1 contributes to Aldara^®^-induced skin inflammation. **(A)** JNK1 level in skins and spleens of *Mapk8^+/+^* and *Mapk8^-/-^* mice **(B)** *Mapk8^+/+^* and *Mapk8^-/-^* mice were shaved and then treated daily with a topical dose of Aldara^®^ cream (62.5 mg/d) for eight consecutive days. Mice were sacrificed 4h after the last application. **(C)** Epidermis thickness (μm) was measured on May Grünwald Giemsa (MGG)-stained sections. Results were collected from two independent experiments (treated, n = 10; untreated, n = 6). **(D)** Representative MGG-stained slides from each experimental group. (scale bar = 50 μm, 200X magnification). **(E)** Quantification of Ki-67+ cells/field. **(F)** Representative Ki67 immunohistochemistry staining from each experimental group. (scale bar = 50 μm, 200X magnification). **(G)** Expression of inflammatory markers in whole skin samples was quantified by RTqPCR. Results were pooled from two independent experiments (treated mice n = 5 and untreated n = 3 per experiment). Results are expressed as median and interquartile range and each dot represents an individual mouse. Statistical analysis was performed using Mann-Whitney test, *p < 0.05, **p < 0.01, ***p < 0.001.

First, we evaluated the JNK1 level in *Mapk8^+/+^* (WT) and *Mapk8^-/-^* skin and spleen and attested the functional invalidation of JNK1 in *Mapk8^-/-^* mice (**Figure 1A**). In order to determine the role of JNK1 signaling pathway in skin inflammation, we evaluated the sensitivity of *Mapk8* ^-/-^ mice to repeated topical applications of Aldara^®^, a classical model that shares many features with human psoriasis (**Figure 1B**). As expected, after 8 days of treatment, WT mice developed clear thickening of the epidermis associated with intense proliferation of basal keratinocytes as revealed by Ki67 staining (**Figures 1C–F**). We did not observe any difference between mock-treated *Mapk8^-/-^* mice and their counterparts but both parameters were significantly reduced after Aldara^®^ treatment (**Figures 1C, D**). Next, we evaluated the expression of psoriasis-associated inflammatory mediators. Aldara^®^ treatment strongly enhanced expression of cytokines (such as *Il17a*, *Il1f6*, *Il19*, *Il1b*, *and Il6*), chemokines (*Cxcl1*) and anti-microbial peptides (*S100a9*) in the skin of *Mapk8^+/+^* mice. Induction of these inflammatory genes was strongly reduced in Aldara^®^-treated *Mapk8^-/-^* mice in comparison to their WT counterpart (**Figure 1G**). These results indicate that JNK1 represents an important signaling pathway in the context of Aldara^®^-induced skin inflammation.

### JNK1 Is Necessary for IL-1β Production Induced by Aldara^®^

**Figure 2:**
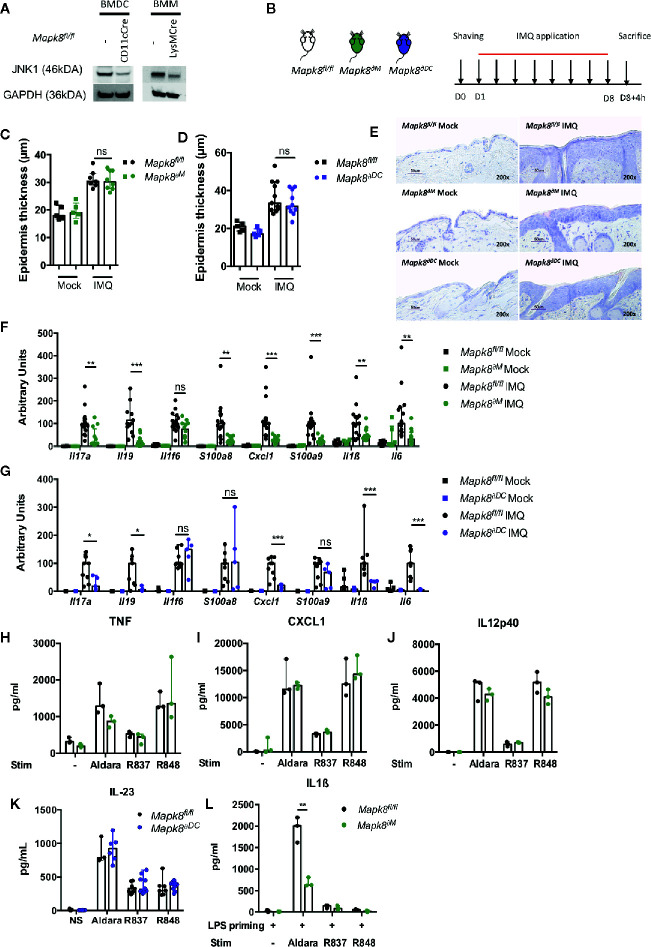
JNK1 is necessary for inflammasome activation by Aldara^®^ cream but not upon TLR7 or TLR7/8 stimulation. **(A)** JNK1 expression in *Mapk8^fl/fl^*, CD11cCre+ *Mapk8^fl/fl^* BMDCs and LysMcre+ *Mapk8^fl/f l^* BMMs. **(B)** *Mapk8^fl/fl^*, *Mapk8^∂M^* and *Mapk8^∂DC^* mice were shaved and then treated daily with a topical dose of Aldara^®^ cream (62.5 mg/d) for eight consecutive days. Mice were sacrificed 4h after the last application. **(C, D)** Epidermis thickness (μm) was measured on MGG-stained sections. Results were pooled and collected from two independent experiments (treated mice n = 7 or 8 or 10 or 11 according to mice groups and untreated n = 5). **(E)** Representative MGG-stained slides from each experimental group. (scale bar = 50 μm, 200X magnification). **(F, G)** Expression of inflammatory markers in whole skin samples was quantified by RTqPCR. Results were pooled from two independent experiments (treated mice n = 5 or 6 or 9 according to mice groups and untreated n = 6). Results are expressed as median and interquartile range and each dot represents an individual mouse. Statistical analysis was performed using Mann-Whitney test, ns, non significant, * p < 0.05, **p < 0.01, ***p < 0.001. **(H)** TNF, **(I)** CXCL1and **(J)** IL12p40 production by BMMs and **(K)** IL-23 production by BMDCs stimulated overnight by Aldara cream (1/2500 dilution in DMSO), R837 (8μg/ml) or R848 (5μg/ml) were quantified by ELISA. **(L)** IL-1β production by BMMs primed for 3h with LPS (100 ng/ml) and then stimulated by Aldara cream (1/100 dilution in DMSO), R837 (8μg/ml) or R848 (10μg/ml) were quantified by ELISA. Results are from one experiment representative of two independent experiments. Results are expressed as median and interquartile range and each dot represents an experimental replicate. Statistical analysis was performed using Mann-Whitney test, **p < 0.01.

Langerhans cells, dermal dendritic cells (DCs) and recruited inflammatory monocytes have been reported to be key contributors to psoriatic plaque formation as sources of IL-23 or IL-1β (13–15). We therefore hypothesized that the production of these Aldara^®^-induced inflammatory cytokines might be dependent on JNK1 expression in myeloid cells. Therefore, we used mice with *Mapk8* floxed gene that express normal level of JNK1 as WT mice and we generated mice lacking JNK1 in the whole myeloid compartment (LysMCre *Mapk8^fl/fl^* mice, targeting monocytes, macrophages and neutrophils: *Mapk8^∂M^*) or in CD11c^+^ cells (**Figure 2A**) (ItgaxCre *Mapk8^fl/fl^*, targeting DCs and Langerhans cells: *Mapk8^∂DC^*). Deletion of JNK1 in either compartment had no effect on epidermal thickening induced by Aldara^®^ treatment (**Figures 2B–E**). Nevertheless, we observed a strong reduction in the expression of many mediators and markers of inflammation in Aldara^®^-treated skins of *Mapk8^∂M^* and *Mapk8^∂DC^* mice compared to *Mapk8^fl/fl^* mice (**Figures 2F, G**).

Next, we evaluated the direct role of JNK1 downstream of the signaling pathway triggered by Aldara^®^ in myeloid cells. For this purpose, we evaluated the *in vitro* cytokine or chemokine production by bone marrow-derived macrophages (BMM) in response to imiquimod (R837, a TLR7 ligand) but also by resiquimod (R848, a TLR7/8 ligand) and Aldara^®^ cream dissolved in DMSO. Levels of TNF, CXCL1 or IL12p40 were similar in BMMs from *Mapk8^∂M^ and Mapk8^fl/fl^* mice, indicating that JNK1 is not critically involved in TLR7-mediated cytokine production in these cells (**Figures 2H–J**). We also evaluated IL-23 production by bone marrow derived dendritic cells (BMDC) and reached similar conclusions (**Figure 2K**). Previous reports indicated that the effect of Aldara^®^ is partially mediated through activation of the inflammasome pathway by isostearic acid, a major component of the vehicle cream (16). We therefore primed BMMs by short-time incubation with LPS (to trigger pro-IL-1β expression) followed by R837, R848, or Aldara^®^ stimulation and measured IL-1β production in the supernatants. We observed robust production of IL-1β upon treatment by Aldara^®^ but not upon R837 or R848 stimulation (**Figure 2L**). In these experimental conditions, JNK1-deficient BMMs produced significantly reduced levels of IL-1β in comparison to WT cells. Taken together, these results indicate that JNK1 expression in the myeloid compartment contributes to Aldara^®^-induced skin inflammation through the IL-1β production system rather than by acting downstream of the TLR7 pathway.

### JNK1 Is Necessary for Acanthosis Induced by Aldara^®^

**Figure 3:**
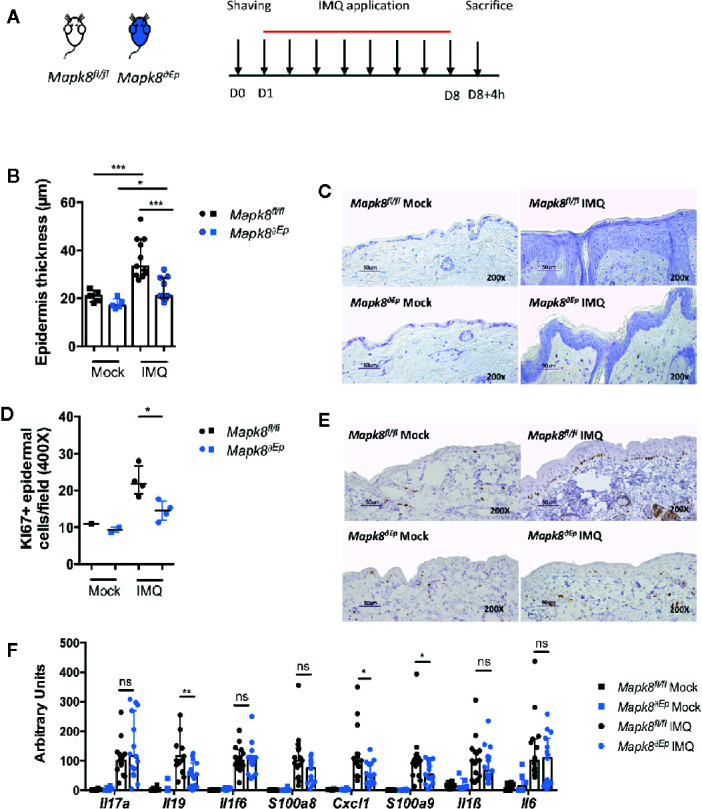
JNK1 is necessary for acanthosis induced by Aldara^®^. **(A)** *Mapk8^fl/fl^* and *Mapk8^∂Ep^* mice were shaved and then treated daily with a topical dose of Aldara^®^ cream (62.5 mg/d) for eight consecutive days. Mice were sacrificed 4 h after the last application. **(B)** Epidermis thickness (μm) was measured on MGG-stained sections. Results are from two independent experiments (treated n = 11 and untreated n = 5). **(C)** Representative MGG-stained slides from each experimental group. (scale bar = 50 μm, 200X magnification). **(D)** Quantification of Ki-67+ cells/field. **(E)** Representative Ki67 immunohistochemistry staining from each experimental group. (scale bar = 50 μm, 200X magnification). **(F)** Expression of inflammatory markers in whole skin samples was quantified by RTqPCR. Results were pooled from two independent experiments (treated n = 15 and untreated n = 6). Results are expressed as median and interquartile range and each dot represents an individual mouse. Statistical analysis was performed using Mann-Whitney test, ns: non significant, *p < 0.05, **p < 0.01, ***p < 0.001.

Deletion of JNK1 in myeloid or DC compartment led to dampened expression of inflammatory markers but did not influence Aldara^®^-induced epidermal thickening, suggesting that these two pathological phenomena could be dissociated. We therefore evaluated the role of JNK1 in keratinocytes by generating K14Cre *Mapk8* ^fl/fl^ (*Mapk8^∂Ep^)* mice (**Figure 3A**). Histological sections indicated that Aldara^®^-induced acanthosis was somewhat reduced of 36% in *Mapk8^∂Ep^* mice in comparison to *Mapk8^fl/fl^* mice (**Figures 3B, C**). This acanthosis decrease is similar to the one observed between the *Mapk8^-/-^* and *Mapk8^+/+^* mice (28%). This was associated with reduced proliferation of basal keratinocytes as revealed by Ki67 staining (**Figures 3D, E**). In sharp contrast, expression of inflammatory genes was found to be comparable in both groups excepted for *Il19*, *Cxcl1* and *S100a9* (**Figure 3F**). These results indicate that JNK1 in epidermal cells contributes to Aldara^®^-induced acanthosis and has a limited involvement in the induction of inflammatory markers.

### Acanthosis Induced by IL-23/IL-17A is Independent of JNK1

**Figure 4:**
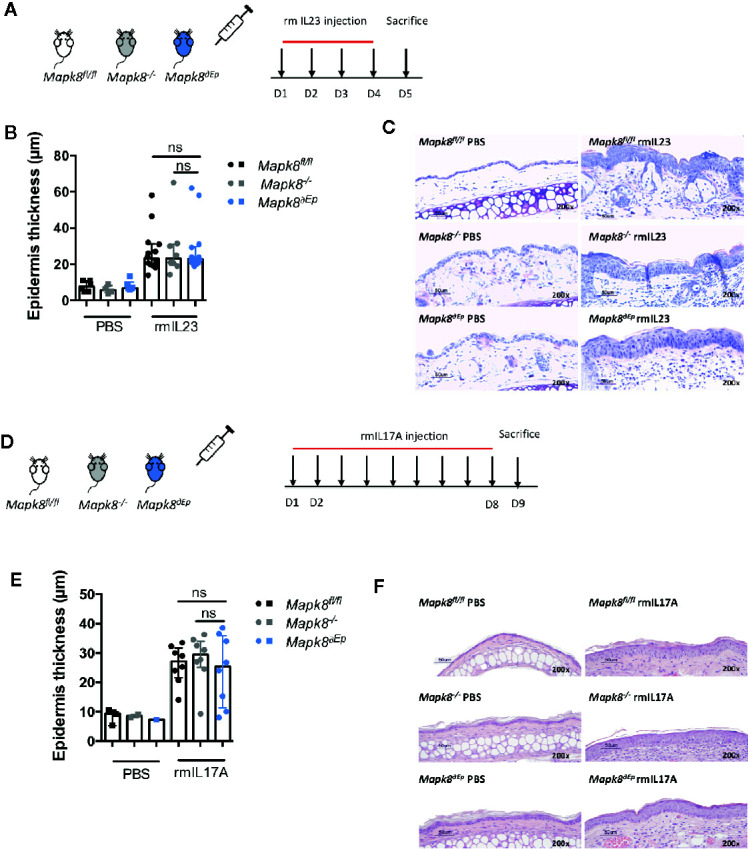
Acanthosis induced by IL23 or IL17A does not require JNK1. **(A)** *Mapk8^fl/fl^*, *Mapk^∂Ep^* and *Mapk8^-/-^ mice* received daily ear injections of rmIL-23 for four consecutive days. Mice were sacrificed 24 h after the last injection. **(B)** Epidermis thickness (μm) was measured on MGG-stained sections. Results are from two independent experiments (rmIL-23 n = 8 or 14 according to mice groups and PBS n = 6 or 4). **(C)** Representative MGG-stained slides from each experimental group. (scale bar = 50 μm, 200X magnification). **(D)** *Mapk8* ^fl/fl^, *Mapk* ^∂Ep^ and *Mapk8* ^-/-^ mice received daily ear injections of rmIL-17A for four consecutive days. Mice were sacrificed 24 h after the last injection. **(E)** Epidermis thickness (μm) was measured on MGG-stained sections. Results are from two independent experiments (rmIL-17 n = 8 and PBS n = 3 or 2 or 1 according to mice groups). **(F)** Representative hematoxylin and eosin (HE)-stained slides from each experimental group. (scale bar = 50 μm, 200X magnification). Results are expressed as median and interquartile range and each dot represents an individual mouse. Statistical analysis was performed using Mann-Whitney test, ns, non significant.

IL-17 signaling in keratinocytes contributes to psoriatic inflammation (17). We hypothesized that the dominant effect of JNK1 on Aldara^®^-induced acanthosis could be related to its role downstream of the IL-17R in epidermal cells, as suggested in patients harboring the *MAPK8* variant (6). We first evaluated the contribution of JNK1 upon repeated intradermal injection of recombinant IL-23 that mediates its effect through induction of IL-17 and IL-22 (18). These experiments were performed in *Mapk8^-/-^*, *Mapk8^∂Ep^*, and *Mapk8^fl/fl^* mice (**Figure 4A**). Recombinant mouse IL-23 induced epidermis thickening to a similar extent in the three groups (**Figures 4B, C**). Next, we injected recombinant mouse IL-17A and reached the same conclusions (**Figures 4D–F**). These results suggest that the dominant role of JNK1 in the Aldara^®^ model is not related to its effect downstream of the IL-17R.

### Transcriptomic and Epigenomic Profiles of Epidermal Cells Upon Aldara^®^ Treatment

**Figure 5:**
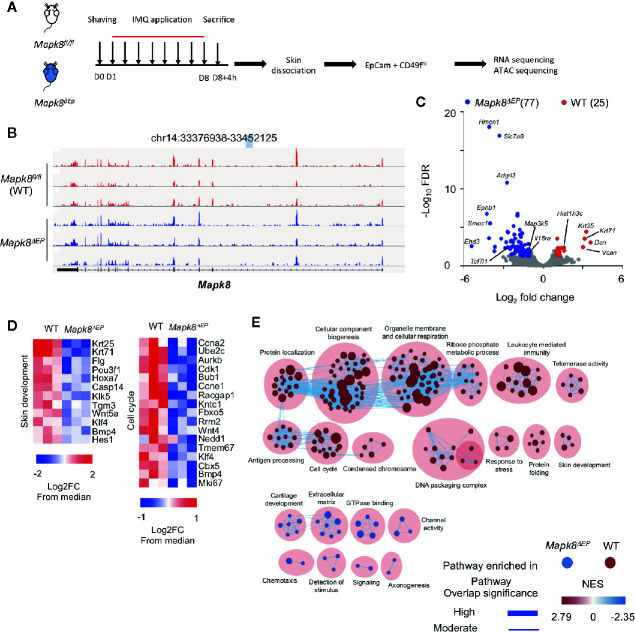
JNK1 signaling contributes to the transcriptomic program of keratinocytes isolated from Aldara^®^-treated mice. **(A)** WT and *Mapk8^∂Ep^* mice were shaved and then treated daily with a topical dose of Aldara^®^ cream (62.5 mg/d) for eight consecutive days. Mice were sacrificed 4h after the last application. After skin dissociation and EpCam+CD49fhi cells isolation, RNA sequencing and ATAC-sequencing were performed. **(B)** Integrative Genomics Viewer tracks showing reads coverage for RNA expression of *Mapk8* gene in WT (red) and *Mapk8^∂EP^* (blue). Gene position is indicated at the top of the panel. **(C)** Volcano plot of RNA-seq data of keratinocytes from WT versus *Mapk8^∂Ep^* mice shows the adjusted P-value versus fold-change (up in WT, red; up in Mapk8^∂Ep^, blue). The numbers of differentially expressed genes are indicated. **(D)** Heatmaps of RNA-seq data comparing the z-score (log2 fold-change (FC) from median) of selected genes involved in pathways that are enriched in WT cells in comparison to *Mapk8^∂EP^* keratinocytes. **(E)** Gene set enrichment network displays clusters of pathways overrepresented in WT (red) and *Mapk8^∂Ep^* (blue) keratinocytes, respectively. Nodes represent gene sets and edges represent mutual overlap. Overlap significance is indicated by the edge’s thickness. Color density indicates NES (Normalized enrichment score).

To further elucidate the role that JNK1 plays in keratinocytes in the context of Aldara^®^-treated skin, we sorted EpCam^+^CD49f^hi^ epidermal cells from WT and *Mapk8^∂Ep^* mice after 8 days of topical application of Aldara^®^ (**Figure 5A**) (gating strategy **Supplementary Figure 1**). We then performed transcriptomic (RNA-Seq) and epigenomic (ATAC-Seq) analysis to define their molecular profiles.

We validated that the *Mapk8* gene gave rise to a truncated mRNA upon K14-driven expression of the Cre recombinase (**Figure 5B**). Then, we identified 102 differentially expressed genes (25 down and 77 up-regulated genes) in JNK1-deficient cells as compared to their controls (FdR<0.01, FC>2) (**Figure 5C**). Several genes involved in epidermal differentiation, proliferation or formation of the cornified envelope such as *Krt25*, *Krt71*, *Casp14*, *Klk5*, *Tgm3*, *Klk5*, *Flg*, and histone-encoding genes were down-regulated in absence of JNK1, suggesting that this kinase controls part of the differentiation program induced in the context of skin inflammation. Geneset enrichment analysis (GSEA) confirmed this notion, as pathways involved in cell cycle, biogenesis or skin development were downregulated in absence of *Mapk8* (**Figures 5D, E**).

**Figure 6:**
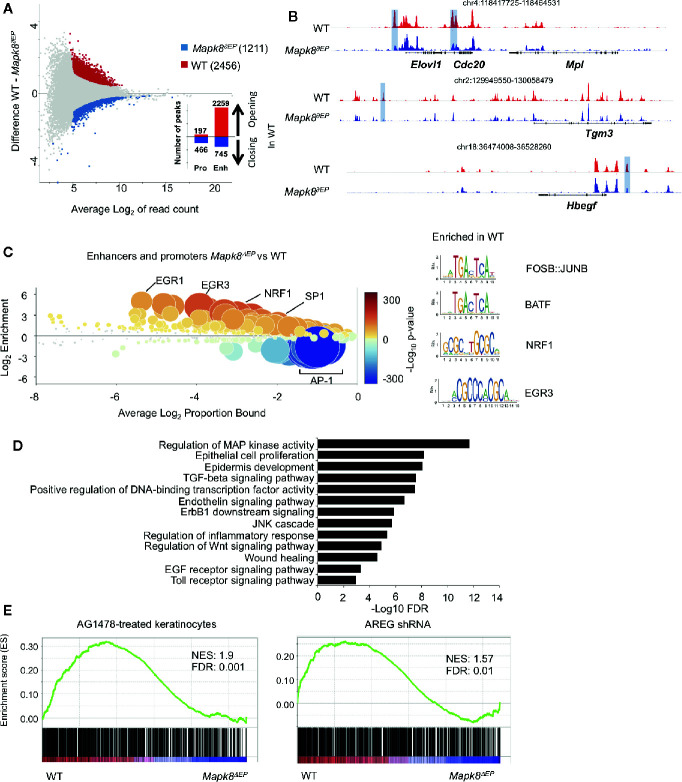
JNK1 drives the epigenomic program of keratinocytes upon Aldara^®^ treatment. **(A)** MA plot of mean ATAC-seq counts per peak showing the differentially open regions of *Mapk8^∂Ep^* keratinocytes (blue) and WT keratinocytes (red) with the indicated number of regions. Histograms indicate the number of opening or closing regions in WT in comparison to *Mapk8^∂Ep^* keratinocytes at promoters and enhancers. **(B)** Representative ATAC-Seq tracks showing differentially accessible regions at the loci of *Elovl1*, *Cdc20*, *Tgm3*, and *Hbegf* genes (highlighted in purple). Position of each region in the genome is indicated at the top of each track. **(C)** CiiiDER analysis of putative transcription factors motifs from differentially open regions of keratinocytes in *Mapk8^∂Ep^* and WT. Transcription factors are colored according to the p-value of their gene coverage and whether they are over- (red) or under- (blue) represented in Mapk8^∂Ep^ keratinocytes. The size of each point is also proportional to log10 P-value. Consensus sequence of FOSB : JUNB, BATF, NRF1 and EGR3 transcription factors are shown with their respective p-values. **(D)** GREAT analysis of pathways putatively regulated by differentially open regions of WT keratinocytes. The adjusted p-values are shown after -Log10 conversion. **(E)** GSEA plots of RNA-Seq data sets and the indicated gene sets. NES and FDR are shown. ATAC-seq was performed on two independent samples from each group.

To further determine the underlying molecular processes at play, we analyzed epigenomic landscapes of these cells by ATAC-Seq approaches. This technique allows us to map open chromatin regions throughout the genome (19). We observed extensive modifications in *Mapk8* deficient cells as 2,456 and 1,211 regions were found to be significantly more or less accessible in controls, respectively (**Figure 6A**). Most of the differentially accessible peaks were located in enhancers rather than in promoters. These observations suggest important and widespread functional impact of JNK1 signaling on the epigenetic programming of epidermal cells during inflammation. For example, we observed decreased accessibility in regulatory elements associated with the genes that encodes *Elovl1*, involved in epidermal barrier formation (20), *Cdc20*, an essential regulator of cell division, *Tgm3*, a transglutaminase involved in the formation of the cornified cell envelope upregulated in psoriasis (21), *Hbegf* (encoding one of the EGFR ligands) (**Figure 6B**). We scanned for binding motifs at the center of ATAC peaks located in these differentially accessible regions using Ciiider algorithm (http://ciiider.com/). We observed a very strong enrichment for consensus binding motifs characteristic of AP-1 family in WT-specific enhancers, strongly supporting the notion that JNK1 acts upstream of c-Jun in this context. (**Figure 6C**). Next, we performed gene-ontology analysis using GREAT (genomic regions enrichment of annotations tool) (22). The most relevant pathways were associated with regions that were less accessible in JNK1-deficient cells. As expected, many of these regions were involved in MAP kinase activity, epithelial cell proliferation and epidermis development. Importantly, several signaling pathways, such as EGFR, TLR and TGFβR were also identified (**Figure 6D**). As EGFR pathway is altered in psoriatic lesions (23), we further investigated this pathway. To specifically define whether our transcriptomic data were compatible with a role of JNK1 downstream of EGFR pathway, we performed GSEA analysis using public datasets from keratinocytes treated with the EGFR tyrosine kinase inhibitor AG1478 or with shRNA targeting amphiregulin (AREG) (**Figure 6E**) (24, 25). Genes that were decreased in both conditions were also significantly depleted in JNK1-deficient keratinocytes. Taken together, these data indicate that JNK1 participates in the differentiation program of epidermal cells in response to inflammatory signals and suggest a potential involvement of the EGFR pathway in this process.

### JNK1 Contributes to Acanthosis Induced by Recombinant AREG

**Figure 7:**
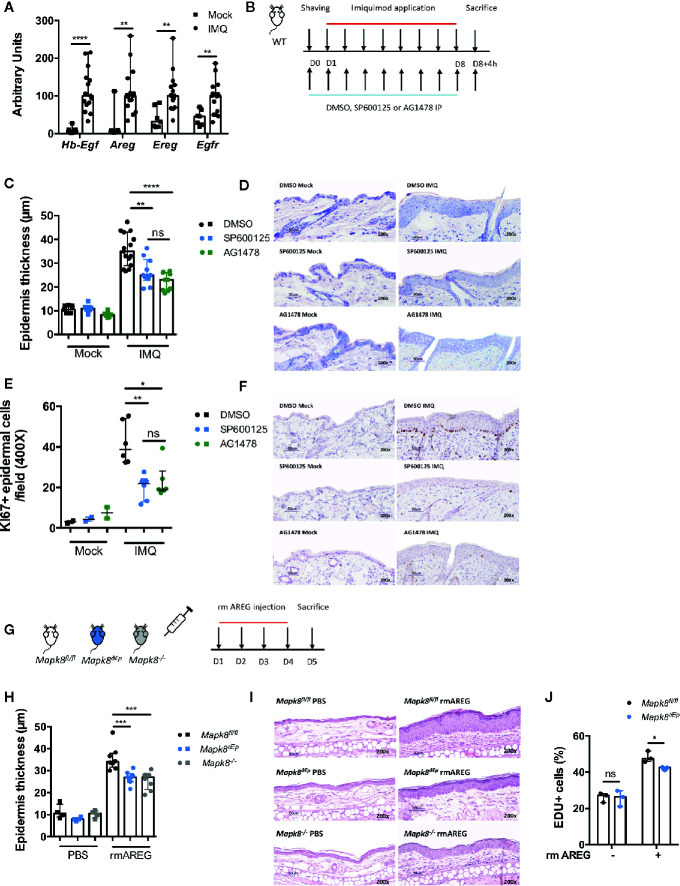
The role of JNK1 in the EGFR axis. **(A)** Expression of EGFR ligands and EGFR receptor in whole skin samples of WT mice stimulated by Aldara^®^ cream for eight consecutive days was quantified by RTqPCR. Results were pooled from two independent experiments. **(B)** WT mice received daily intraperitoneal injection of DMSO, SP600125 or AG1478, then mice were shaved and treated daily with a topical dose of Aldara^®^ cream (62.5 mg/d) for eight consecutive days. Mice were sacrificed 4h after the last application. **(C)** Epidermis thickness (μm) was measured on MGG-stained sections. Results were collected from two independent experiments (treated n = 15 or 10 and untreated n = 9 or 6 according to mice groups). **(D)** Representative MGG-stained slides from each experimental group. (scale bar = 50 μm, 200X magnification). **(E)** Quantification of Ki-67+ cells/field. **(F)** Representative Ki67 immunohistochemistry staining from each experimental group. (scale bar = 50 μm, 200X magnification). **(G)** *Mapk8^fl/fl^*, *Mapk^∂Ep^* and *Mapk8^-/-^* mice received daily ear injections of rmAREG for four consecutive days. Mice were sacrificed 24h after the last injection. **(H)** Epidermis thickness (μm) was measured on MGG-stained sections. Results are from one experiment representative of two independent experiments (rmAREG n = 8 or PBS n = 4 per experiment). **(I)** Representative HE-stained slides from each experimental group. (scale bar = 50 μm, 200X magnification). **(J)** Living proliferating murine primary keratinocytes after 48h of rmAREG (100ng/ml) stimulation. Results are from one experiment representative of two independent experiments. Results are expressed as median and interquartile range and each dot represents an individual mouse. Statistical analysis was performed using Mann-Whitney, ns: non significant, *p < 0.05, **p < 0.01, ***p < 0.001,****p < 0.0001.

**Figure 8:**
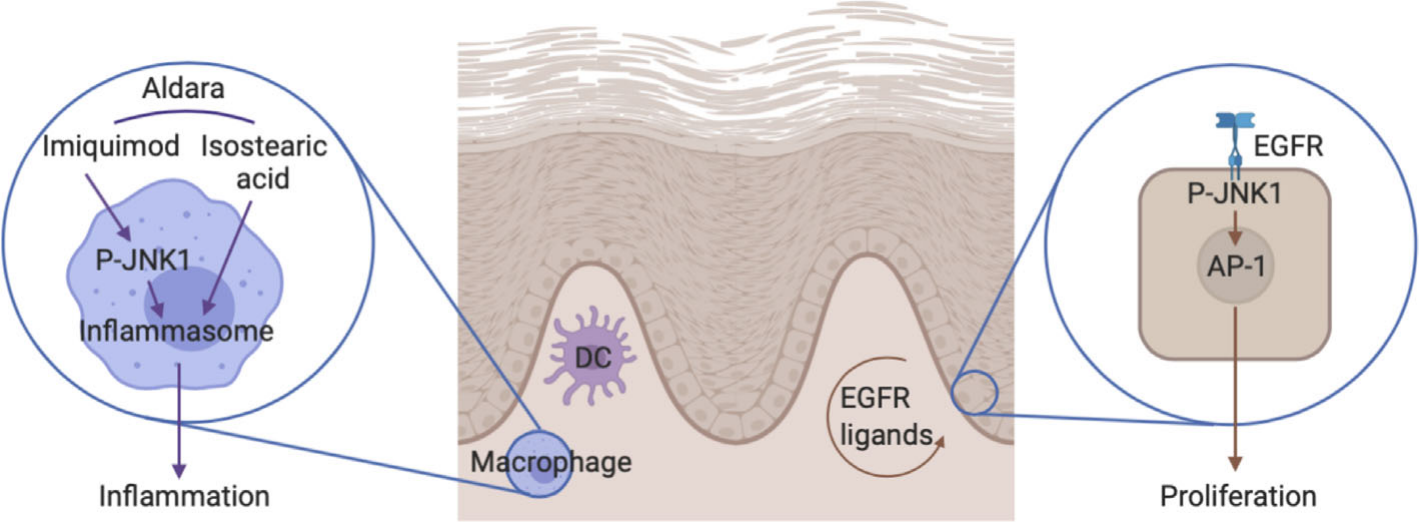
The role of JNK1 in the Aldara^®^-induced skin inflammation. On one hand, JNK1 contributes to IL-1β production by macrophages in the context of inflammasome activation by Aldara^®^ cream. On the other hand, JNK1 contributes to keratinocyte proliferation by acting downstream of the EGFR.

Based on our epigenomic data, we postulated that during Aldara^®^-induced inflammation and psoriasis, JNK1 could act downstream of the EGFR pathway in epidermal cells. As previously described (26, 27) and consistent with human data (23, 28, 29), we observed that expression of EGFR ligands such as Hb-EGF, AREG, EREG and of EGFR was increased in the skin of Aldara^®^-treated compared to untreated mice (**Figure 7A**). Therefore, to assess the potential role of EGFR signaling in this model, WT mice received intraperitoneal injections of AG1478, an EGFR tyrosine kinase inhibitor or DMSO (**Figure 7B**). In parallel, WT mice were treated with SP600125, a classical JNK inhibitor. We noticed that Aldara^®^-induced epidermal thickening (**Figures 7C, D**) and keratinocyte proliferation (Ki-67 staining) (**Figures 7E, F**) were reduced in WT mice receiving SP600125 or AG1478 compared to DMSO-treated mice. Henceforth, to further assess the role of JNK1 downstream of the EGFR signaling, we assessed epidermal thickening in response to recombinant mouse AREG (rmAREG) intradermal injections in *Mapk8^-/-^*, *Mapk8^∂Ep^* and *Mapk8^fl/fl^* mice (**Figure 7G**). Histological sections revealed reduced acanthosis in *Mapk8^-/-^* and *Mapk8^∂Ep^* treated mice compared to *Mapk8^fl/fl^* controls (**Figures 7H, I**). Finally, we evaluated the proliferation of murine primary keratinocytes stimulated by rmAREG *in vitro* for 48 hours and observed a significant reduction in EDU+ cells in keratinocytes isolated from *Mapk8^∂Ep^* mice in comparison to their *Mapk8^fl/fl^* littermates (**Figure 7J**). These data indicate that JNK1 contributes to keratinocyte proliferation downstream of the EGFR pathway, thereby participating to Aldara^®^-induced acanthosis (**Figure 8**).

## Discussion

JNKs (JNK1, 2, and 3) are named after their capacity to phosphorylate and activate the protein c-Jun, a member of the AP-1 family of transcription factors. However they have multiple other targets and also function as a transcriptional co-regulator (30). JNK1 is ubiquitously expressed and contributes to inflammation in multiple settings. For instance, initial studies indicated that JNK1 contributed to Th2 differentiation and function (31, 32). In the context of experimental autoimmune encephalomyelitis, JNK1 in myeloid cells was shown to participate in the induction of pathogenic Th17 responses (33). Furthermore, in experimental arthritis, this kinase regulates macrophage migration (34). In contrast, JNK1 plays a deleterious role for the control of systemic *Candida albicans* infection through its effect on CD23 expression by innate immune cells (35). Herein, we demonstrate that it also participates in Aldara^®^-induced skin inflammation. We show that JNK1 in myeloid cells was required to trigger the expression of inflammatory cytokines. Imiquimod is a TLR7 ligand that directly activates dermal dendritic and Langerhans cells (36, 37). However, the presence of isostearic acid in the Aldara^®^-cream also participates in the full inflammatory response through activation of the inflammasome pathway (38). *In vitro* experiments with bone marrow-derived macrophages indicated that JNK1 was involved in the inflammasome pathway rather than downstream of TLR signaling (**Figure 8**). This is in line with previous data revealing that JNK1 directly phosphorylates NLRP3, thereby promoting caspase-1 activation and IL-1β processing (39). It is possible that JNK2 partially compensate for the lack of JNK1 as previously demonstrated in the context of obesity-related inflammation or epidermis development (40, 41). Importantly, despite the fact that inflammatory markers were clearly decreased in mice lacking JNK1 in myeloid cells, epidermal thickening was not affected. We therefore hypothesized that JNK1 could also play a direct role in keratinocytes. We observed that epidermal proliferation induced by Aldara^®^ treatment was decreased in *Mapk8^∂Ep^* mice. As heterozygous *MAPK8* mutation in patients suffering from chronic mucocutaneous *C. albicans* was associated with decreased *in vitro* responsiveness to IL-17A (6), we evaluated the capacity of recombinant IL-23 or IL-17A to trigger acanthosis in these mice. Our data indicate that JNK1 activation downstream of the IL-17R does not play a dominant role for the induction of cell proliferation in this experimental setting. Our transcriptomic and epigenomic data on epidermal cells from Aldara^®^-treated *Mapk8^∂Ep^* mice indicated that JNK1 signaling was involved in key biological processes linked to cell proliferation and keratinocyte differentiation. Among the potential upstream regulators, we identified the EGFR system, which represents a critical regulator of skin inflammatory responses. This is consistent with the observation that *Jnk1^-/-^Jnk2^+/-^* or *cJun^∂EP^* mice display impaired embryonic eyelid closure, a feature regulated by the EGFR axis (5, 42, 43). Multiple evidences suggest the involvement of this pathway in psoriasis (44). Indeed, EGFR-ligands such as TGF-α, HB-EGF and AREG are more present in psoriatic skin compared to healthy skin. EGFR expressed mostly by basal keratinocytes is not upregulated in psoriatic lesions (28, 45). Moreover, EGFR kinases inhibitors used as cancer therapy such as Erlotinib, Lapatinib and monoclonal antibodies targeting the extracellular domain of EGFR including Cetuximab were shown to improve psoriatic lesions in cancer patients (46–50). We observed that EGFR kinase inhibitor AG1478 also decreased Aldara^®^-induced acanthosis, suggesting that this pathway is also involved in this experimental setting. Taken together, our results suggest that acanthosis induced by Aldara^®^ requires both EGFR and IL-17 signaling. In this context, the effect we observed in absence of JNK1 or upon pharmacological inhibition appears to be limited to the EGFR pathway.

Our results open a promising new therapeutic window stemming from our description in a mouse model of the role of JNK1 in psoriasis. Developing pharmaceutical drug inhibitors for clinical use remains a challenging task, as witnessed by adverse effects after an oral JNK inhibitor treatment for idiopathic pulmonary fibrosis (51). Nonetheless, the local use of a JNK inhibitor for the treatment of acute hearing loss showed a favorable safety profile (52). Moreover, the majority of psoriatic patients suffers from mild-to-moderate psoriasis that can be managed by topical treatments (53, 54). These elements constitute a rationale for the clinical evaluation of topical JNK inhibitors for the treatment of mild-to-moderate cutaneous forms of psoriasis.

## Data Availability Statement

The data presented in the study are deposited in the GEO repository, accession number GSE164581.

## Ethics Statement

The animal study was reviewed and approved by The Institutional animal care and user committee from the IBMM (Institut de Biologie et de Médecine Moléculaire) (protocol number CEBEA-IBMM-2013-70).

## Author Contributions

AL conducted most of the experiments. AA contributed to some experiments. ST, NI, and MN provided technical help for the experiments. AA performed bioinformatics analysis. AL, AA, and SG analyzed the data and prepared the figures. SG supervised the work and wrote the manuscript. All authors were involved in critically revising the manuscript for important intellectual content. All authors had full access to the data and approved the manuscript before. The article was submitted by the corresponding author. All authors contributed to the article and approved the submitted version.

## Funding

The Fonds National de la Recherche Scientifique (FRS-FNRS, Belgium), the European Regional Development Fund (ERDF) of the Walloon Region (Wallonia-Biomed portfolio, 411132-957270) and the “Actions de Recherches Concertées” (AV.12/17) supported this study. SG is a senior research associate of the FRS-FNRS. A.L. is supported by a grant from the FRIA-FNRS and the Fond ERASME.

## Conflict of Interest

The authors declare that the research was conducted in the absence of any commercial or financial relationships that could be construed as a potential conflict of interest.
