# Supplementary material for: JNK1 Signaling Downstream of the EGFR Pathway Contributes to Aldara®-Induced Skin Inflammation
Source: Front Immunol. 2021 Feb 5;11:604785. doi: 10.3389/fimmu.2020.604785 (PMC7892463; doi:10.3389/fimmu.2020.604785)
Supplement: Supplementary file 1 [file Table_1.docx]

Supplementary Material

# 1 Supplementary Table

| Name | Primer FW | Primer RV | Probe |
| --- | --- | --- | --- |
| *Actb* | TCCTGAGCGCAAGTACTCTGT | CTGATCCACATCTGCTGGAAG | ATCGGTGGCTCCATCCTGGC |
| *Il17a* | GCTCCAGAAGGCCCTCAG | CTTTCCCTCCGCATTGACA | ACCTCAACCGTTCCACGTCACCCTG |
| *Il19* | CAGGAGCATTAAGCCTGGAG | CTCTCCTGATGGTCCTGGAA | TGCTGCATGACCAACAACCTGC |
| *Il1f6* | GTGTGGATCCTGCAGAACAA | GGCATGGGAGCAAGGTAATA | TGCAGTCCCAAGGAAAGAGCAAACA |
| *S100a8* | CCTTTGTCAGCTCCGTCTTC | CAAGGCCTTCTCCAGTTCAG | AAGGAAATCTTTCGTGACAATGCCG |
| *S100a9* | AGCCTTGAGCAAGAAGATGG | TTGATGGAAGGTGTCGATGA | TGGAGCGCAGCATAACCACCA |
| *Cxcl1* | CCGAAGTCATAGCCACACTC | TTTCTGAACCAAGGGAGCTT | AAGGCAAGCCTCGCGACCAT |
| *Il1b* | CAACCAACAAGTGATATTCTCCATG | GATCCACACTCTCCAGCTGCA | CTGTGTAATGAAAGACGGCACACCCACC |
| *Il6* | GAGGATACCACTCCCAACAGACC | AAGTGCATCATCGTTGTTCATACA | CAGAATTGCCATTGCACAACTCTTTTCTCA |
| *Hb-egf* | TCTGGCCGCAGTGTTGTCC | GGTTTGTGGATCCAGTGGGAG |  |
| *Areg* | GCTGAGGACAATGCAGGGTAA | GTGACAACTGGGCATCTGGA |  |
| *Ereg* | TGCTTTGTCTAGGTTCCCACC | GGCGGTACAGTTATCCTCGG |  |
| *Egfr* | ACCTCTCCCGGTCAGAGATG | CTTGTGCCTTGGCAGACTTTC |  |
