# Supplementary figures and images for: JNK1 Signaling Downstream of the EGFR Pathway Contributes to Aldara®-Induced Skin Inflammation
Source: Front Immunol. 2021 Feb 5;11:604785. doi: 10.3389/fimmu.2020.604785 (PMC7892463; doi:10.3389/fimmu.2020.604785)

## Supplemental data

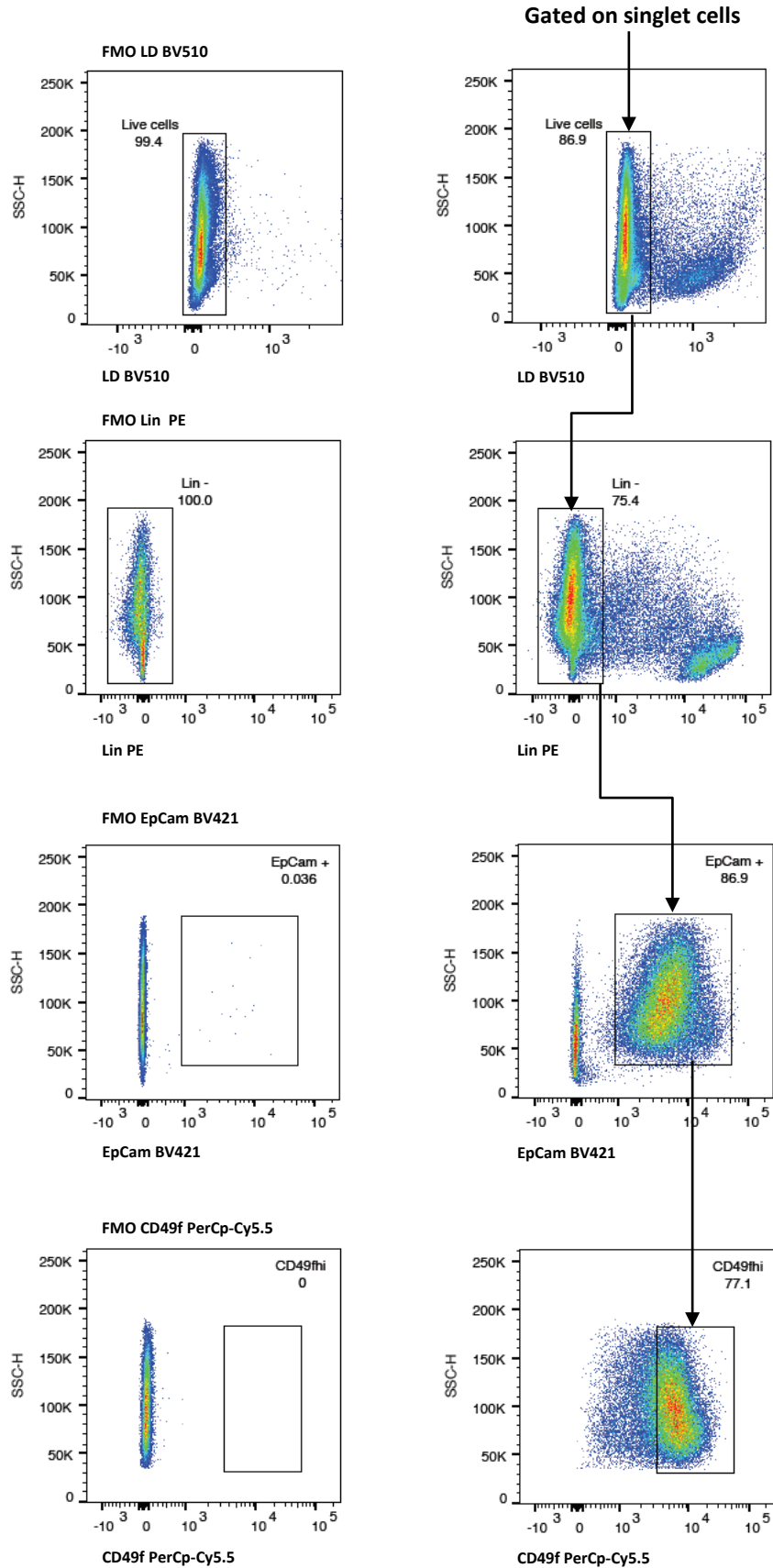

Supplement: Supplementary file 2 [file Image_1.pdf]
